# Bias-Adjusted Three-Step Multilevel Latent Class Modeling with Covariates

**DOI:** 10.1080/10705511.2023.2300087

**Published:** 2024-02-16

**Authors:** Johan Lyrvall, Zsuzsa Bakk, Jennifer Oser, Roberto Di Mari

**Affiliations:** a University of Catania; b Leiden University; c Ben-Gurion University

**Keywords:** Bias-adjusted three-step estimation, covariates, latent class analysis, multilevel

## Abstract

We present a bias-adjusted three-step estimation approach for multilevel latent class models (LC) with covariates. The proposed approach involves (1) fitting a single-level measurement model while ignoring the multilevel structure, (2) assigning units to latent classes, and (3) fitting the multilevel model with the covariates while controlling for measurement error introduced in the second step. Simulation studies and an empirical example show that the three-step method is a legitimate modeling option compared to the existing one-step and two-step methods.

## 1. Introduction

Latent class analysis is a model-based approach used to create a clustering of units of analysis on the basis of a set of observed indicator variables. The clustering is expressed in terms of a latent variable with some number of discrete categories, or *latent classes*. For example, latent class analysis has been used to identify repertoires of political participation (Oser, 2022), risk profiles for adolescent substance abuse (Lanza & Rhoades, 2013), and patterns of study strategy (Hickendorff et al., 2010). When the data have a multilevel structure, that is, when lower-level units are nested in higher-level units (for example, students nested in a school, patients nested in a centre), the *multilevel* latent class model (Vermunt, 2003) is used to account for higher-level dependencies in the observed variables. Such non-independence arises because respondents in the same group give more similar answers to each other than respondents from different groups.

Multilevel latent class analysis involves introducing a latent variable at the higher level as a categorical random effect defining the distribution of the lower-level clusters within groups. For example, Henry & Muthén (2010) identified a typology of adolescent smokers, non-, moderate, and heavy smokers, and found that the types can be clustered into two community-level segments: low-use and high-use communities. Fagginger Auer et al. (2016) identified four study strategies among students, which could be clustered into four types of teachers with different probabilities of eliciting these study strategies.

Usually, the research interest in latent class analysis lies in explaining latent class membership by covariates, or external variables (for example, neighborhood effects on school performance). Identifying the latent classes (measurement model) is then only the first step of the analysis, after which a more complex model that includes the covariates (structural model) is specified. In most applications, the focus is on the lower-level latent classes and covariates are included only on this level.

In single-level latent class analysis, different approaches for estimation of the covariate effects are available, namely the classical one-step, or simultaneous, approach (Lazarsfeld & Henry, 1968), the two-step approach (Bakk & Kuha, 2018) and the different three-step approaches. The seminal works on three-step LCA were put forth by Vermunt (2010) and Bolck et al. (2004). Extensions include three-step LCA for distal outcomes (Bakk et al., 2013; Lanza et al., 2013) and for latent Markov models (Di Mari et al., 2016). The general recommendation is to use stepwise methods (Asparouhov & Muthén, 2014).

Using the one-step approach, both the measurement and structural model are estimated simultaneously to obtain maximum-likelihood estimates. This approach is apparently natural, however it has serious defects (see e.g., Asparouhov & Muthén, 2014; Bakk & Kuha, 2018; Vermunt, 2010). The full model needs to be re-estimated when a change is made to only one part of it, e.g., increase or decrease in the number of classes, inclusion or exclusion of covariates in the structural model.

Practically, re-estimating the full model can be computationally demanding, especially when the number of potential covariates is large. Furthermore, while the covariates are taken to be class predictors, changes to the structural model can change the latent class solution, especially if underlying model assumptions are violated (Bakk & Kuha, 2018; Masyn, 2017; Vermunt, 2010). This can occur to the extent that comparisons of different estimated structural models become effectively meaningless. In addition, many applied researchers see the introduction of covariates as a separate step after the classification model has been constructed. Different research groups may even build different structural models on the same measurement model.

To avoid the problems of the one-step approach, stepwise methods separate the estimation of the measurement model from the estimation of the structural model. Using the naive three-step approach, (1) the measurement model is estimated alone, (2) units are assigned to latent classes and (3) posterior class assignments are related to the covariates. This was a popular approach as it avoids the defects of the one-step approach, however it introduces a problem of its own: classification error in the second step, yielding bias in the step-3 estimates of the covariate effects (Bolck et al., 2004).

In the last 20 years, many developments have been suggested for single-level latent class analysis to address this issue. The bias-adjusted three-step methods correct the bias by explicitly modeling the classification error in the second step (Bakk et al., 2013; Bolck et al., 2004; Vermunt, 2010). Using the two-step approach (Bakk & Kuha, 2018), (1) the measurement model is estimated alone and (2) the full model is estimated with the measurement parameters held fixed at their step-1 estimates. In the two-step approach, the structural model is estimated conditional on measurement model parameter estimates from step 1, and no actual clustering step is performed.

While the one-step approach can be considered the statistical benchmark in bias and efficiency compared to the stepwise estimators (Bakk et al., 2013; Bakk & Kuha, 2018; Bolck et al., 2004; Vermunt, 2010), applied researchers tend to prefer using the bias-adjusted three step approach, because of the practical appeal of working with an explicit dependent variable. However, while extensions to multilevel latent class models exist for both the one-step approach (Vermunt, 2003) and the two-step approach (Di Mari et al., 2023), the bias-adjusted three-step approach exists only for single-level latent class models. As such, there is a lack of modeling options for multilevel latent class analysis with covariates with respect to single-level latent class analysis with covariates.

In the current paper, we fill this important gap by introducing a bias-adjusted three-step approach for multilevel latent class analysis. The proposed method is a multilevel extension of the bias-adjusted three-step ML approach for single-level LCA of Vermunt (2010). Our contribution complements the existing set of methodologies for the multilevel context with the typically preferred analytical approach in applied social research. Since the research interest in applied latent class analysis typically lies in the association between covariates and the lower-level latent classification, the proposed method was developed for structural modelling on this level. A similar extension has been developed previously for latent Markov modeling with covariates (Di Mari et al., 2016).

The remainder of the paper is outlined as follows. First we introduce the multilevel latent class model with covariates. Then, we discuss the bias-adjusted three-step ML approach for this model, deriving the correction under standard model assumptions. Subsequently, we report the results of a simulation study in which we compare the method to the one- and two-step methods under different conditions. We next present an empirical application wherein we identify citizenship norms among adolescents and analyze its association with socioeconomic status. The article ends with a summary of the main results and possible directions for future research.

## 2. The Multilevel Latent Class Model

Let $Y_{\text{ij}}=(Y_{\text{ij}1},\ldots ,Y_{\text{ijH}})$ be a vector of observed responses, where *Y_ijh_* denotes the response of low-level unit (individual) $i=1,\ldots ,n_{j}$ in high-level unit (group) j=1,…,J on the *h*-th categorical indicator variable (item) (Vermunt, 2003). For simplicity of exposition, we focus on dichotomous indicators with values 0 and 1. Let $Y_{j}=(Y_{1j},\ldots ,Y_{n_{J}j})$ be the set of responses for all low-level units *i* in high-level unit *j*. The $Y_{j}$ for different *j* are taken to be independent.

Let *W_j_* be a categorical latent class (LC) variable defined at the higher level, with possible, mutually exclusive values m=1,…,M and probabilities $\omega_{m}=P(W_{j}=m)>0.$ Given a realization of *W_j_*, let *X_ij_* be a categorical LC variable defined at the low level with possible, mutually exclusive values t=1,…,T and conditional probabilities $\pi_{t|m}=P(X_{\text{ij}}=t|W_{j}=m)>0.$ The *X_ij_* for the same *j* are taken to be conditionally independent given *W_j_*, that is,

(1) $$P(X_{\text{ij}},\ldots ,X_{n_{j}j})=\sum_{m=1}^{M}P(W_{j}=m)\prod_{i=1}^{n_{j}}P(X_{\text{ij}}=t|W_{j}=m).$$

The simple LC model defines the following probability structure on $Y_{\text{ij}},$

(2) $$P(Y_{\text{ij}})=\sum_{t=1}^{T}P(X_{\text{ij}}=t)P(Y_{\text{ij}}|X_{\text{ij}}=t).$$

The probability of observing a particular response pattern $P(Y_{\text{ij}})$ is a linear combination of *T* class-specific item response probabilities $P(Y_{\text{ij}}|X_{\text{ij}}=t),$ where the (unconditional) class proportions $P(X_{\text{ij}}=t)$ serve as weights. Furthermore, we make the local independence assumption that the *H* indicator variables are conditionally independent within the *X_ij_*, so that

(3) $$P(Y_{\text{ij}}|X_{\text{ij}}=t)=\prod_{h=1}^{H}P(Y_{\text{ijh}}|X_{\text{ij}}=t)=\prod_{h=1}^{H}\phi_{h|t}^{Y_{\text{ijh}}}{\left(1-\phi_{h|t}\right)}^{1-Y_{\text{ijh}}},$$

where $\phi_{h|t}=P(Y_{\text{ijh}}=1|X_{\text{ij}}=t).$ For simplicity of notation, we express the local independence assumption in terms of the second equality, leading to the following specification of the simple LC model,

(4) $$P(Y_{\text{ij}})=\sum_{t=1}^{T}P(X_{\text{ij}}=t)\prod_{h=1}^{H}P(Y_{\text{ijh}}|X_{\text{ij}}=t).$$

To account for the multilevel structure of the data, the multilevel LC model of Vermunt (2003) defines the following probability structure on $Y_{\text{ij}},$

(5) $$P(Y_{\text{ij}})=\sum_{m=1}^{M}\omega_{m}\sum_{t=1}^{T}\pi_{t|m}\prod_{h=1}^{H}P(Y_{\text{ijh}}|X_{\text{ij}}=t).$$

The probability of observing a particular response pattern $P(Y_{\text{ij}})$ is a linear combination of *M* high-level class-specific simple LC models, where the class proportions *ω_m_* serve as weights. This is the so-called non-parametric modeling approach (for the parameteric approach, wherein the higher-level latent variable is continuous, see, e.g., Asparouhov & Muthén, 2014). As can be seen, we make the common assumption that the item response probabilities do not directly depend on *W_j_*, that is, $P(Y_{\text{ij}}|X_{\text{ij}}=t,W_{j}=m)=P(Y_{\text{ij}}|X_{\text{ij}}=t)$ (Vermunt, 2003).

The multilevel LC model can be defined in terms of logistic equations. For *ω_m_* we can consider the following nonparametric random-effect model,

(6) $$P(W_{j}=m)=\frac{\;\text{exp}\;(\alpha_{0m})}{1+\sum_{l=2}^{M}\;\text{exp}\;(\alpha_{0l})},$$

while for $\pi_{t|m},$

(7) $$P(X_{\text{ij}}=t|W_{j}=m)=\frac{\;\text{exp}\;(\gamma_{0\text{tm}})}{1+\sum_{s=2}^{T}\;\text{exp}\;(\gamma_{0\text{sm}})},$$

and for $\phi_{h|t},$

(8) $$P(Y_{\text{ijh}}=1|X_{\text{ij}}=t)=\frac{\;\text{exp}\;(\beta_{t}^{h})}{1+\;\text{exp}\;(\beta_{t}^{h})},$$

where the parameters for the first classes and response categories are set to zero for identification purposes, that is, $\alpha_{01}=\gamma_{01m}=\beta_{1}^{h}=0.$ We denote the vector of parameters of the measurement model for the items *Y_ijh_* by $\theta_{1}=(\phi_{1|1},\ldots ,\phi_{H|T}).$ A logistic parametrization of the simple LC model can be obtained similarly on the basis of these equations. For $P(X_{\text{ij}}=t),$ the *m*-subscripts can be omitted from Equation (7), while, for $\phi_{h|t},$ Equation (8) can be used directly.

Covariates can be included in the multilevel LC model to predict class membership. Let $Z_{j}^{H}$ be a high-level covariate, $Z_{\text{ij}}^{L}$a low-level covariate, and $Z_{\text{ij}}=(Z_{j}^{H},Z_{\text{ij}}^{L}).$ To predict high-level and low-level class membership, we can extend the logistic equations as follows,

(9) $$P(W_{j}=m|Z_{j}^{H})=\frac{\;\text{exp}\;(\alpha_{0m}+\alpha_{1m}Z_{j}^{H})}{1+\sum_{l=2}^{M}\;\text{exp}\;(\alpha_{0l}+\alpha_{1l}Z_{j}^{H})}$$

(10) $$P(X_{\text{ij}}=t|W_{j}=m,Z_{\text{ij}})=\frac{\;\text{exp}\;(\gamma_{0\text{tm}}+\gamma_{1\text{tm}}Z_{\text{ij}}^{L}+\gamma_{2\text{tm}}Z_{j}^{H})}{1+\sum_{s=2}^{T}\;\text{exp}\;(\gamma_{0\text{sm}}+\gamma_{1\text{sm}}Z_{\text{ij}}^{L}+\gamma_{2\text{sm}}Z_{j}^{H})},$$

with $\alpha_{11}=\gamma_{11m}=\gamma_{21m}=0$ for identification. We collect the structural model parameters in the vector by $\gamma =(\gamma_{011},\ldots ,\gamma_{2\text{TM}}).$ Under these parametrizations we can define the multilevel LC model for $Y_{\text{ij}}|Z_{\text{ij}}$ as

(11) $$P(Y_{\text{ij}}|Z_{\text{ij}})=\sum_{m=1}^{M}P(W_{j}=m|Z_{j}^{H})\sum_{t=1}^{T}P(X_{\text{ij}}=t|W_{j}=m,Z_{\text{ij}})\prod_{h=1}^{H}P(Y_{\text{ijh}}|X_{\text{ij}}=t),$$

where we further assume that the observed response patterns $Y_{\text{ij}}$ are conditionally independent of the covariates $Z_{\text{ij}}$ given low-level class membership *X_ij_* and high-level class membership *W_j_*. This conditional independence assumption is standard in the multilevel LCA literature (Bakk et al., 2022; Di Mari et al., 2023). The equivalent conditional independence assumption in single-level LC modelling is standard as well (e.g. Bakk & Kuha, 2018).

In applied multilevel LCA with covariates the research interest typically lies in the lower-level structural model for *X_ij_*. Then, covariates $Z_{j}^{H}$ may be excluded, or included to control for variation at the higher level, while the substantive research questions regard the effect of $Z_{\text{ij}}^{L}$ on *X_ij_*. For example, Bijmolt et al. (2004) fit a multilevel LC model for international consumer segmentation in financial product ownership, excluding predictors of country-level segments and including demographic variables as predictors of individual-level segments.

## 3. Selecting the Numbers of Latent Classes on Lower and Higher Level

The description of the multilevel LC model with covariates above takes the numbers of LCs on lower and higher level as given. In applied LCA, selecting these values is often a distinct exercise. It is generally recommended to carry out this task on the model without the covariates, and then hold the selected numbers of classes fixed when the covariates are added (Masyn, 2017). To do so, two approaches are typically used, namely the sequential and the simultaneous approaches.

The sequential approach of Lukočienė et al. (2010) involves a hierarchical three-step model fitting procedure. First, a set of simple LC models with different numbers of LCs (*T*) are fitted and the optimal number is selected. Second, this value is held fixed and a set of multilevel LC models with different numbers of high-level LCs (*M*) are fitted, selecting the best candidate. Third, the selected *M* is held fixed and model selection is done again at the lower level, to determine the final *T*.

The more direct simultaneous approach involves estimating the multilevel LC model for all combinations of the different values of *T* and *M* of interest. In both model selection approaches, the optimal values of *T* and *M* can be selected with standard information criteria, such as the BIC. This can be combined with measures of class separation, such as the entropy-based R^2^ (Magidson, 1981), which can be defined at both the lower level and the higher level (Lukočienė et al., 2010).

For a detailed discussion about information criteria and likelihood-based tests for model selection in LC analysis, see Nylund et al. (2007) (see also Collins et al., 1993). When the selection of indicators is an issue, see the approaches proposed by Bartolucci et al. (2016) and by Dean & Raftery (2010).

## 4. Three-Step Estimation of the Multilevel Latent Class Model

In this section we describe a bias-adjusted three-step ML estimation approach for the multilevel LC model, taking the number of LCs on lower level and higher level as given. The procedure involves (i) estimating the simple LC model without covariates, (ii) estimating class membership and classification error, and (iii) estimating a logistic regression model for LC membership while correcting for the classification error introduced in step 2. As such, the proposed method is a multilevel extension of the bias-adjusted three-step ML approach for single-level LCA of Vermunt (2010).

### 4.1. Step 1 - Estimating the Multilevel Measurement Model

In the first step, the LC model without covariates is estimated by maximum likelihood (ML). The model of interest is the multilevel LC model in Equation (5), which the researcher may identify by means of model selection, using for example the sequential approach (Lukočienė et al., 2010) or the simultaneous approach.

In step 1 the simple LC model is fitted, ignoring the multilevel structure. Parameter estimates can be obtained by maximizing the following log-likelihood function

(12) $$l_{\text{step}1}=\sum_{i=1}^{N}\;\text{log}\;\left[\sum_{t=1}^{T}P(X_{\text{ij}}=t)\prod_{h=1}^{H}P(Y_{\text{ijh}}|X_{\text{ij}}=t)\right],$$

**Figure 1.**
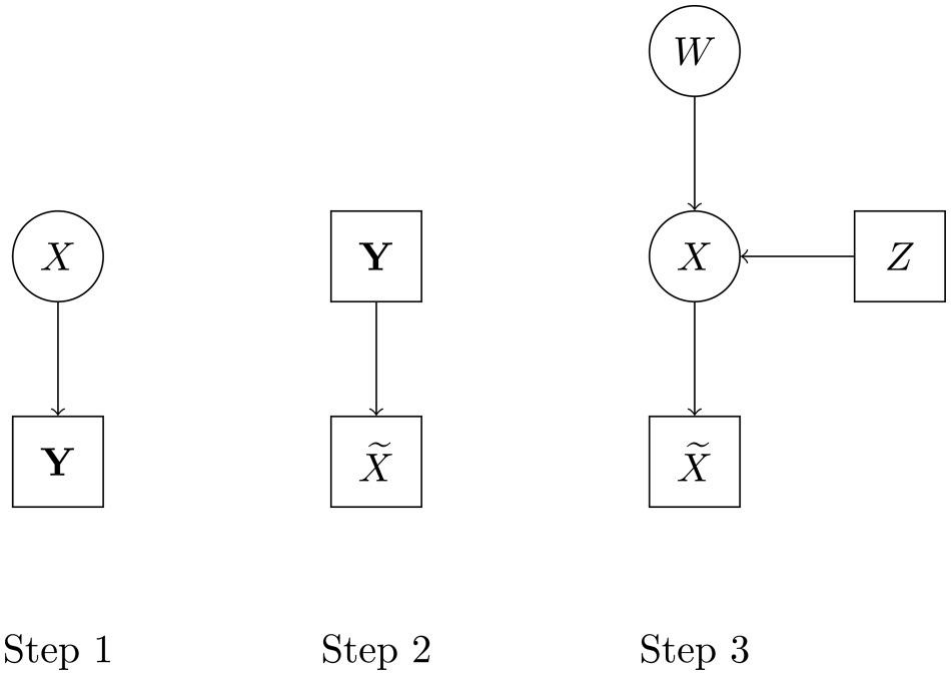
The steps of the proposed three-step approach.

We graphically summarize the first step in the left-most panel of Figure 1.

### 4.2. Step 2 - Posterior Classification and Classification Error

Given the step-1 parameter estimates, the posterior LC membership probability $P(X_{\text{ij}}=t|Y_{\text{ij}})$ can be obtained using Bayes’ rule (Goodman, 1974a, 1974b; Hagenaars, 1992; MacLahlan & Peel, 2000) in the following manner

(13) $$P(X_{\text{ij}}=t|Y_{\text{ij}})=\frac{P(X_{\text{ij}}=t)P(Y_{\text{ij}}|X_{\text{ij}}=t)}{P(Y_{\text{ij}})}.$$

From this equation, individual *i* can be assigned to the LC *t* on the basis of different classification rules, the most common of which are modal assignment and proportional assignment.

Let ${\tilde{X}}_{\text{ij}}$ denote the estimated class membership of individual *i* in group *j*. Modal assignment yields a hard partitioning in which *i* is allocated weight $P({\tilde{X}}_{\text{ij}}=s|Y_{\text{ij}})=1$ if the posterior membership probability $P(X_{\text{ij}}=s|Y_{\text{ij}})$ is the largest, and weight zero otherwise. Proportional assignment yields a soft (crisp) partitioning in which *i* is allocated weight $P({\tilde{X}}_{\text{ij}}=s|Y_{\text{ij}})=P(X_{\text{ij}}=s|Y_{\text{ij}}).$ Another classification rule is random assignment, which yields a hard partitioning by assigning *i* to the ${\tilde{X}}_{\text{ij}}$ that is randomly drawn from the distribution $P({\tilde{X}}_{\text{ij}}=s|Y_{\text{ij}})$ (Goodman, 2007). In most applications the preferred rule is modal assignment because it minimizes classification error (see e.g., Bakk et al., 2013; Vermunt, 2010).

Regardless of which classification rule is used, the assigned class will differ from the true class for some units (Bolck et al., 2004; Hagenaars, 1990). The overall quality of the classification can be quantified by the expected proportion of classification error $P({\tilde{X}}_{\text{ij}}=s|X_{\text{ij}}=t),$ which expresses the probability of assignment to a certain class conditional on the true class membership. This quantity can be computed as a weighted average over all possible response patterns,

(14) $$\begin{array}{cc} 20P({\tilde{X}}_{\text{ij}}=s|X_{\text{ij}}=t) & =\sum_{Y}P(Y|X_{\text{ij}}=t)P({\tilde{X}}_{\text{ij}}=s|Y)\\ & =\frac{\sum_{Y}P(Y)P(X_{\text{ij}}=t|Y)P({\tilde{X}}_{\text{ij}}=s|Y)}{P(X_{\text{ij}}=t)}. \end{array}$$

When the number of unique response patterns is very large, it is convenient to replace the weights P(Y) with their empirical distribution, leading to

(15) $$P({\tilde{X}}_{\text{ij}}=s|X_{\text{ij}}=t)=\frac{\frac{1}{N}\sum_{j=1}^{J}\sum_{i=1}^{n_{j}}P(X_{\text{ij}}=t|Y_{\text{ij}})P({\tilde{X}}_{\text{ij}}=s|Y_{\text{ij}})}{P(X_{\text{ij}}=t)}.$$

The second step is summarized graphically in the mid panel of Figure 1.

### 4.3. Step 3 - Estimating the Multilevel Structural Model

The starting point of the correction methods for three-step LC analysis is that unadjusted estimation of the relationship between ${\tilde{X}}_{\text{ij}}$ and $Z_{\text{ij}}$ causes bias toward zero in the covariate effects of interest (Bolck et al., 2004). Given the step-2 LC assignments ${\tilde{X}}_{\text{ij}}$ and the expected frequency of classification error $P({\tilde{X}}_{\text{ij}}=s|X_{\text{ij}}=t),$ we can obtain unbiased coefficient estimates defining the relationship between the unobservable LC variable *X_ij_* and the covariates $Z_{\text{ij}}.$

Consider the joint distribution of *W*, *X*, **Y**, $\tilde{X}$ given **Z**. Given the assumptions of Equation (11), that **Y** is conditionally independent of **Z** and *W* given *X*, and that $\tilde{X}$ only depends on **Y**, this quantity can be decomposed as

(16) $$P(W,X,Y,\tilde{X}|Z)=P(W|Z^{H})P(X|W,Z)P(Y|X)P(\tilde{X}|Y).$$

From this equation it is possible to derive the conditional distribution $P(\tilde{X}|Z)$ by marginalising over lower- and higher-level LCs, and by summing over response patterns, which yields

(17) $$\begin{array}{cc} P(\tilde{X}|Z) & =\sum_{W}\sum_{X}\sum_{Y}P(W|Z^{H})P(X|W,Z)P(Y|X)P(\tilde{X}|Y)\\ & =\sum_{W}P(W|Z^{H})\sum_{X}P(X|W,Z)\sum_{Y}P(Y|X)P(\tilde{X}|Y)\\ & =\sum_{W}P(W|Z^{H})\sum_{X}P(X|W,Z)\frac{\sum_{Y}P(X|Y)P(Y)P(\tilde{X}|Y)}{P(X)}\\ & =\sum_{W}P(W|Z^{H})\sum_{X}P(X|W,Z)P(\tilde{X}|X). \end{array}$$

The first equality follows directly from Equation (16), and the second equality re-arranges the summations. The third equality re-writes the last term based on the equation for the expected proportion of classification error. This was defined in the right-hand side of Equation (14). The fourth equality simplifies this term into the notation for the expected proportion of classification error. This is the multilevel version of the derivation for the three-step approach in single-level LC modeling (Vermunt, 2010) and latent Markov modeling (Di Mari et al., 2016). As can be seen, the last right-hand side of the last equality of Equation (17) is similar to the equation for the multilevel LC model with covariates, but with the step-2 estimates for the expected proportion of classification error in the role of the response probabilities P(Y|X).

Unbiased estimates of the structural parameters *γ* and *α* can be obtained by estimating the right-hand side of Equation (17) as a multilevel LC model with covariates, with the low-level class assignments ${\tilde{X}}_{\text{ij}}$ as a single indicator with known error probabilities $P({\tilde{X}}_{\text{ij}}|X_{\text{ij}}).$ This involves maximizing the log-likelihood function

(18) $$l_{\text{step}3}=\sum_{j=1}^{J}\;\text{log}\;\sum_{m=1}^{M}P(W_{j}=m|Z_{j}^{H})\prod_{i=1}^{n_{j}}\sum_{t=1}^{T}P(X_{\text{ij}}=t|W_{j}=m,Z_{\text{ij}})P({\tilde{X}}_{\text{ij}}=s|X_{\text{ij}}=t).$$

This is similar to the second step of the two-step approach, but with the classification error probabilities in the place of the item-specific response probabilities $\prod_{h=1}^{H}P(Y_{\text{ijh}}|X_{\text{ij}}=t).$

Step 3 is summarized graphically in the right-most panel of Figure 1.

## 5. Simulation Study

### 5.1. Design

We conduct a simulation study to assess the quality of the proposed bias-adjusted three-step ML approach when the model is correctly specified. As classification rule we use modal assignment, which is the most common in LCA applications and minimizes classification error. The method is compared to the one-step and two-step approaches, which are the currently available modeling options for multilevel latent class analysis with covariates.

We evaluate the relative performance of the three-step estimator based on bias and variation in the estimated covariate effects. The finite-sample quality of stepwise estimators for LCA has been found to depend on class separation and sample size (Bakk & Kuha, 2018; Di Mari et al., 2023; Vermunt, 2010). In multilevel LCA, both lower-level and higher-level class separation affect the finite-sample behaviour (Lukočienė et al., 2010).

As population model we use the multilevel LC model with 3 lower-classes *X*, 2 higher-level classes *W*, and 10 binary indicator variables *Y*. Higher-level class proportions are $P(W_{j}=1)=0.6$ and $P(W_{j}=2)=0.4.$ We consider one continuous lower-level covariate *Z* generated from the standard normal distribution. The population slope parameters $\gamma_{1\text{tm}}$ are set to $\gamma_{121}=\gamma_{131}=-0.25$ and $\gamma_{122}=\gamma_{132}=0.25.$ Additionally, we consider population slope parameters equal to zero, $\gamma_{121}=\gamma_{131}=\gamma_{122}=\gamma_{132}=0,$ however, we discuss this in a more limited investigation of statistical power in the results. The first class *X* = 1 has high probability to score 1 on all items, the second class has high probability to score 1 on the last 5 items and 0 on the first 5 items, and class *X* = 3 has high probability to score 0 on all items.

Lower-level class separation is manipulated via the item-response probabilities. In the small, moderate and large separation conditions, the probabilities of the most likely response are 0.7, 0.8 or 0.9, respectively. At the higher level we manipulate class separation via the random intercepts, such that the expected conditional class proportions in the moderate separation condition are equal to $P(X_{\text{ij}}=1|W_{j}=1)=0.29,P(X_{\text{ij}}=2|W_{j}=1)=0.33,$ $P(X_{\text{ij}}=3|W_{j}=1)=0.38$ and $P(X_{\text{ij}}=1|W_{j}=2)=0.38,$ $P(X_{\text{ij}}=2|W_{j}=2)=0.33,P(X_{\text{ij}}=3|W_{j}=2)=0.29,$ while in the large separation condition they are equal to $P(X_{\text{ij}}=1|W_{j}=1)=0.14,P(X_{\text{ij}}=2|W_{j}=1)=0.32,P(X_{\text{ij}}=3|W_{j}=1)=0.54$ and $P(X_{\text{ij}}=1|W_{j}=2)=0.60,P(X_{\text{ij}}=2|W_{j}=2)=0.25,$ $P(X_{\text{ij}}=3|W_{j}=2)=0.15.$ For the sample size, we use 100 or 500 lower-level units and 30, 50 or 100 higher-level units.

We quantify class separation as the average entropy-based R^2^ (Lukočienė et al., 2010; Magidson, 1981) across the 500 random samples, which is one of the most commonly used measures of class separation in the social sciences. The entropy-based R^2^ expresses how much the prediction of class membership improves when using the information on the responses: a value equal to 0 corresponds to no separation and a value equal to 1 corresponds to perfect separation. The average higher-level R^2^-entropy for the samples with the moderate and large higher-level separation conditions are 0.76 (0.57 when the lower-level sample size is equal to 100 and 0.96 when the lower-level sample size is equal to 500) and 1.00, respectively. The average lower-level R^2^-entropy for the samples with the small, moderate, and large lower-level separation conditions are 0.51, 0.80, and 0.96, respectively.

From each of the 36 crossed simulation conditions (see Table 1) we generate 500 random samples. Our simulation setting is similar to previous studies on multilevel LCA (Di Mari et al., 2023; Lukočienė et al., 2010). Data generation and model estimation is carried out using the R package multilevLCA (Lyrvall et al., 2023)^1^.

**Table 1.** Simulation conditions with crossed combinations of lower-level (LL) and higher-level (HL) sample size and separation for simulation study with 100 replications for each condition.

Condition	Sample size		Separation	
	LL	HL	LL	HL
1	100	30	small	moderate
2	500	30	small	moderate
3	100	50	small	moderate
4	500	50	small	moderate
5	100	100	small	moderate
6	500	100	small	moderate
7	100	30	moderate	moderate
8	500	30	moderate	moderate
9	100	50	moderate	moderate
10	500	50	moderate	moderate
11	100	100	moderate	moderate
12	500	100	moderate	moderate
13	100	30	large	moderate
14	500	30	large	moderate
15	100	50	large	moderate
16	500	50	large	moderate
17	100	100	large	moderate
18	500	100	large	moderate
19	100	30	small	large
20	500	30	small	large
21	100	50	small	large
22	500	50	small	large
23	100	100	small	large
24	500	100	small	large
25	100	30	moderate	large
26	500	30	moderate	large
27	100	50	moderate	large
28	500	50	moderate	large
29	100	100	moderate	large
30	500	100	moderate	large
31	100	30	large	large
32	500	30	large	large
33	100	50	large	large
34	500	50	large	large
35	100	100	large	large
36	500	100	large	large

### 5.2. Results

**Figure 2.**
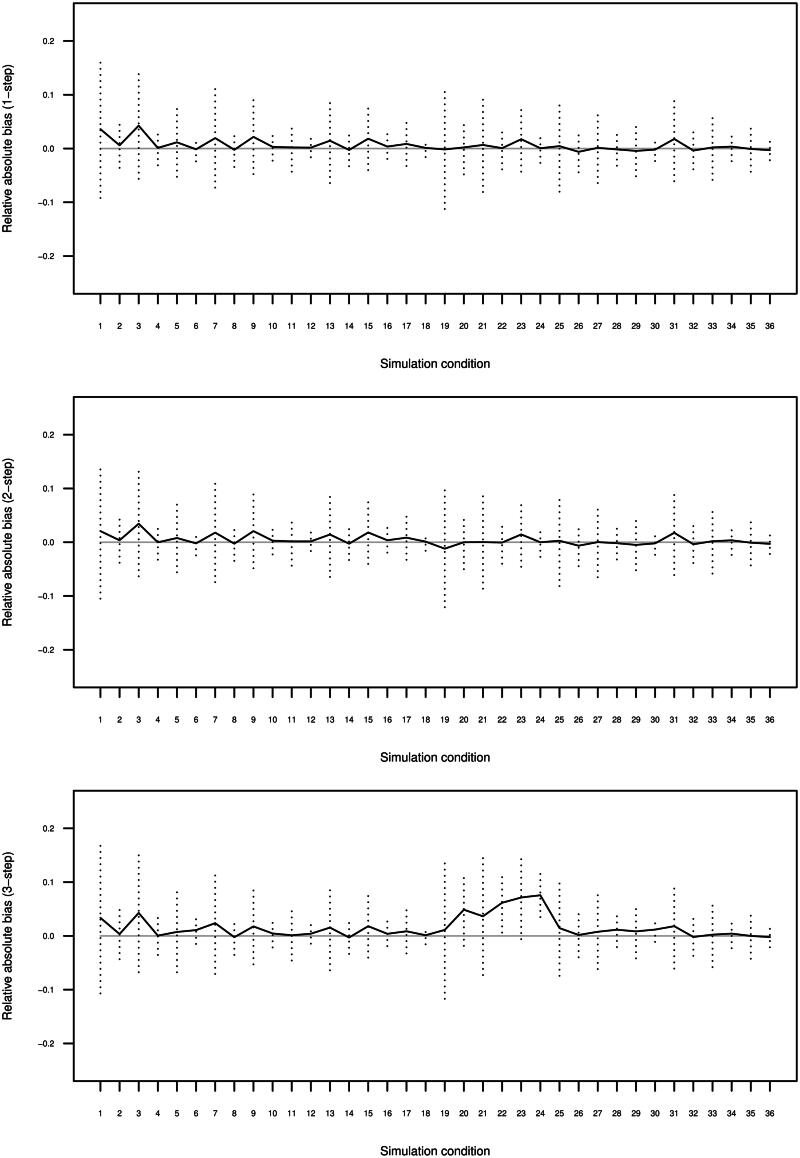
Estimated bias (solid) and their Monte Carlo standard deviation (dotted) for the 500 replications per 36 conditions, averaged across the four different $\gamma_{1\text{tm}},$ separately for the three estimators.

The results are presented averaged across the four slope parameters *γ*_121_, *γ*_131_, *γ*_122_, and *γ*_132_ and the 500 replications for the three-, two-, and one-step estimators. Figure 2 displays average relative absolute bias and efficiency, measured in terms of the Monte Carlo standard deviation of the relative absolute bias. As can be seen, when the degree of higher-level separation is moderate (simulation conditions 1-18) the three-step method performs essentially identical to the one- and two-step methods. In these conditions the performance varies systematically across the two values for the lower-level sample size: the three estimators are less biased and more efficient when the lower-level sample size is 500 (average R ${}_{\text{high}}^{2}$-entropy equal to 0.96) compared to when the lower-level sample size is 100 (average R ${}_{\text{high}}^{2}$-entropy equal to 0.57).

The same systematic variation in efficiency is shown for the three estimators when the higher-level separation is large (simulations conditions 19-36, average R ${}_{\text{high}}^{2}$-entropy equal to 1.00), however, the performance in terms of bias varies across them in these conditions. While the one-step and two-step methods perform comparably, it can be observed that the performance of the three-step method improves when the degree of lower-level separation is larger. Its performance is problematic when the lower-level separation is small (simulation conditions 19-24, average R ${}_{\text{low}}^{2}$-entropy equal to 0.51) - in these situations the alternative methods are preferred. The bias for the three-step estimator is reduced substantially when the lower-level separation is moderate (simulation conditions 25-30, average R ${}_{\text{low}}^{2}$-entropy equal to 0.80) but still slightly higher compared to the one-step estimator and the two-step estimator. The three-step is unbiased when the lower-level separation is large (simulation conditions 31-36, average R ${}_{\text{low}}^{2}$-entropy equal to 0.96).

**Figure 3.**
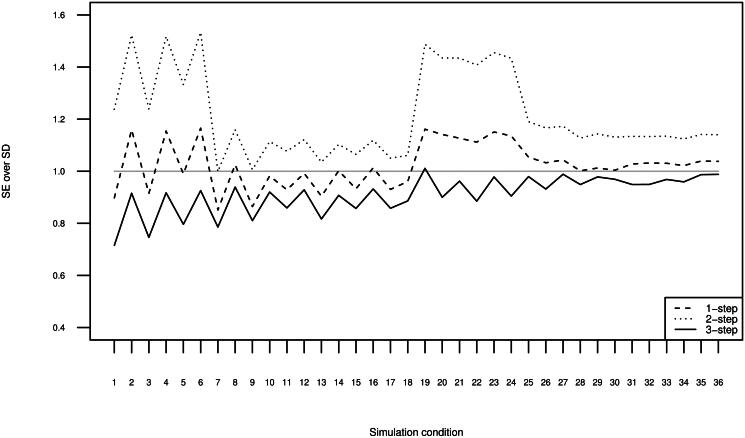
Ratio of estimated standard error to Monte Carlo standard deviation for the 500 replications per 36 conditions, averaged across the four different $\gamma_{1\text{tm}}.$

Figure 3 displays the ratio of the average SE to SD. For the two-step method we use the corrected SEs to account for measurement uncertainty in its first step (the correction is based on pseudo-ML theory and exploits the full variance matrix; see Bakk & Kuha, 2018; Di Mari et al., 2023, for details). The one-step SEs and the three-step SEs are based on the expected information matrix. We first note that the one-step SEs are closest to unbiased in most conditions compared to the SEs for the two-step method and the three-step method, while the two-step SEs are over-estimated in most simulation conditions for the over-correction of the two-step estimator in the single-level context, see Bakk & Kuha, 2018). The three-step SEs are under-estimated, which is in line with previous findings for the single-level context (Bolck et al., 2004; Vermunt, 2010). The downward bias for the three-step method is serious (10-30%) when the higher-level separation is moderate and the lower-level separation is small (simulation conditions 1-6), but is reduced as these conditions improve. When the higher-level separation is large and the lower-level separation is large (simulation conditions 31-36), the under-estimation for the three-step method is slight (up to about 5%).

**Figure 4.**
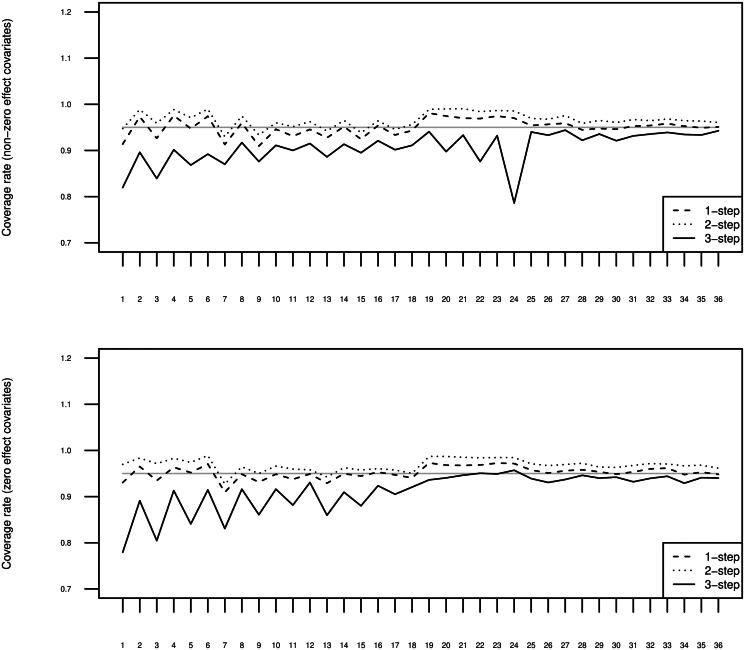
Estimated coverage rates of 95% confidence intervals for the 500 replications per 36 conditions, averaged across the four different $\gamma_{1\text{tm}},$ separately for the simulation study with non-zero covariate effects and the simulation study with zero covariate effects.

The top panel of Figure 4 displays the estimated coverage rates of 95% confidence intervals. The bottom panel displays these values for the samples with population covariate effects equal to zero. The three-step estimator yields undercoverage of the true covariate effects, but this undercoverage is slight (below 5%) when the higher-level separation is large and the lower-level separation is moderate to large (simulation conditions 25-36). When the higher-level separation is large and the lower-level separation is small (simulation conditions 19-24), the performance of the three-step estimator is equally good with zero effect covariates, but problematic with non-zero effect covariates. The problematic performance can be explained by the severe bias in the three-step estimates for the covariate effects in the same conditions. Regardless of the covariate effects, the one-step estimator yields close to correct coverage, while the two-step estimator yields slight overcoverage.

These results are aligned with previous research in single-level LC modeling, in which the bias has been shown to be greater and the efficiency to be lower when the sample size is smaller and the separation is weaker (Bakk & Kuha, 2018; Vermunt, 2010). The present study shows how this performance varies across combinations of separation on the lower level and the higher level in the multilevel LC modeling. How can we explain that the three-step method is sensitive to the lower-level separation when the higher-level separation is large? The explanation lies in the feature that the three-step method involves a classification step wherein the higher-level clustering is ignored, meaning that it does not take into account the information about the hierarchical clustering structure. This loss of information is greater when the higher-level separation is larger. In comparison, the one-step and two-step methods always use the full amount of information on the clustering, making their performance less sensitive to the degree of higher-level separation.

## 6. An Application

To illustrate the proposed bias-adjusted three-step ML method we analyze citizenship norms among adolescents using survey data from the International Civic and Citizenship Education Study, which were collected by the International Association for the Evaluation of Educational Achievement (IEA) in 2016 (Schulz et al., 2018). The survey was conducted in school classes of 14 years old in 22 different areas: Bulgaria, Chile, Colombia, Croatia, Denmark, Dominican Republic, Estonia, Finland, Hong Kong, Italy, Latvia, Lithuania, Malta, Mexico, Netherlands, Norway, Peru, Russia, Slovenia, South Korea, Sweden, and Taiwan. Prior political research has analyzed different waves of the survey to investigate citizenship norms using latent class analysis (Hooghe et al., 2016; Hooghe & Oser, 2015; Oser et al., 2023; Oser & Hooghe, 2013). The data analysis is carried out using the R package multilevLCA (Lyrvall et al., 2023)^2^.

Respondents were presented a variety of citizenship norms and asked to rate each item in terms of what defines a good adult citizen. We combine the answer options “very important” and “quite important” into the value 1, while we combine the answer options “not important at all” and “not very important” into the value 0. The 12-item battery includes obeying the law (*obey*), promoting human rights (*rights*), participating in local activities (*local*), working hard (*work*), supporting activities to protect the environment (*envir*), voting (*vote*), learning about the country’s history (*history*), showing respect for government representatives (*respect*), following political news (*news*), participating in peaceful protest (*protest*), engaging in political conversations (*discuss*), and joining a political party (*party*). Table 2 shows that the means in the pooled sample range from 0.34 to 0.92.

**Table 2.** Means of the 12 dichotomous items in the pooled sample.

Item	Mean
obey	0.92
rights	0.84
local	0.82
work	0.86
envir	0.87
vote	0.83
history	0.82
respect	0.83
news	0.77
protest	0.64
discuss	0.46
party	0.34

Consistent with previous research on the same data (Di Mari et al., 2023), we consider the model T=4,M=3. The separation conditions for this model are rather typical in applied LCA (the R ${}_{\text{low}}^{2}$-entropy is equal to 0.64 and the average proportion of classification error for step-1 model is equal to 0.17). Compared to the simulation study, the estimated class solution exhibits large class separation at the higher level and somewhere between small and moderate separation on the lower level, which are suboptimal conditions for the performance of the three-step. This allows us to see how severely the performance of the proposed estimator are affected by these suboptimal conditions.

Table 3 presents the estimated class solution of this specification. At the lower level, class 1, the “Maximal” adolescent, places importance on all the items; class 2, the “Engaged”, scores somewhat lower on all items, placing little importance on engaging in political conversations or joining a political party; class 3, the “Duty”, emphasizes the traditional items but not the more self-expressive items; class 4, “Subject”, has low probabilities to assign importance to all the items. At the higher level, class 1 (50%) has the highest relative frequency of “Engaged” adolescents, while in class 2 (41%) the majority of youth are “Maximal”, and class 3 (9%) has the highest conditional probability for “Duty” students. In all the country-level classes, “Subject” is the least prevalent.

**Table 3.** Class proportions on lower level (LL) and higher level (HL) and class-specific response probabilities for the multilevel measurement model.

	LL Class 1	LL Class 2	LL Class 3	LL Class 4
	(Maximal)	(Engaged)	(Duty)	(Subject)
Class proportion				
HL Class 1 (0.5000)	0.3292	0.4676	0.1584	0.0448
HL Class 2 (0.4091)	0.5714	0.2803	0.1190	0.0293
HL Class 3 (0.0909)	0.2064	0.2842	0.4783	0.0312
Response probability				
obey	0.9788	0.9217	0.8793	0.4080
rights	0.9819	0.9456	0.4364	0.1337
local	0.9698	0.9145	0.4243	0.1391
work	0.9368	0.8450	0.7824	0.3820
envir	0.9834	0.9622	0.5251	0.2207
vote	0.9699	0.7477	0.7803	0.1768
history	0.9461	0.7939	0.6825	0.2175
respect	0.9341	0.7969	0.7926	0.1844
news	0.9603	0.6586	0.7021	0.0908
protest	0.8632	0.5885	0.2959	0.1107
discuss	0.7925	0.2079	0.2816	0.0229
party	0.5983	0.1303	0.2440	0.0209

Considering the estimated high-class membership, we can see a geographical pattern. Most of the Nordic-European, Eastern European, and South American countries belong to class 1: Bulgaria, Estonia, Finland, Latvia, Lithuania, Malta, Norway, Slovenia, Sweden, Chile, and Colombia. The countries that belong to class 2 are the Mediterranean-European, North American, Central American, and Asian countries and one South American country: Hong Kong, Russia, South Korea, Taiwan, Dominican Republic, Mexico, Italy, Croatia, and Peru. The Continental Northern European countries belong to class 3: Denmark and the Netherlands.

We now investigate the association between lower-level class membership and maternal education using the proposed bias-adjusted three-step ML approach for multilevel LC models and compare the results to the one- and two-step approaches. The covariate is a binary indicator that takes on the value 1 if the mother has post-high school education level (50% of the respondents), and 0 otherwise (50%). In step 2, respondents are assigned to latent classes by means of modal assignment, since this is the most commonly used assignment rule in applied LCA. Table 4 shows that the (lower-level) classes do not differ much in their probability of assignment to the wrong class. The lower-level entropy-based R^2^ is equal to 0.64 and the higher-level entropy-based R^2^ is equal to 1, which, based on the simulation results, are sub-optimal conditions. On the basis of the simulation results, we can therefore expect a larger difference across the estimated covariate effects between the three-step method and its alternatives than between the one-step method and the two-step method.

**Table 4.** Estimated misclassification probabilities for step 2 of the three-step approach, with *X* the true class and $\tilde{X}$ the assigned class.

	*X* = 1	*X* = 2	*X* = 3	*X* = 4
	(Maximal)	(Engaged)	(Duty)	(Subject)
$\tilde{X}=1$	0.8702	0.1381	0.0235	0.0000
$\tilde{X}=2$	0.1156	0.7817	0.1363	0.0059
$\tilde{X}=3$	0.0142	0.0794	0.8142	0.1022
$\tilde{X}=4$	0.0000	0.0008	0.0260	0.8920

Table 5 presents the estimated covariate effects on lower-level class membership. “Maximal” is the reference class and “high school or less” is the reference level of the covariate. As expected, the three-step slope estimates differ more from the slope estimates for the one-step method and the two-step method compared to the difference between the alternative estimators. Nevertheless, substantively, the results for three approaches are well aligned. When maternal education is higher the probability of belonging to the “Engaged” class relative to the “Maximal” class is smaller, with the greatest effect size in country-level class 3. We can see a similar positive effect, but at a greater magnitude, with respect to the probability of belonging to the “Subject” class relative to the “Maximal” class. It is substantial in country-level class 3. The effect of maternal education on the probability of belonging to the “Duty” class relative to the “Maximal” class is rather weak. The precise point estimates for the three-step estimator are in line with the direction of the effects and the relative effect sizes. We can also see that the difference in the one-step SEs and the two-step SEs is smaller than what we would expect given the simulation results. As such, the pattern of significance of the covariate effects is overall comparable.

**Table 5.** Estimated effect of maternal post-high school education on latent class membership for the three-, two-, and one-step estimators, where class 1 (maximal) is the reference category, *** *p*-value < 0.01, ** *p*-value < 0.05, * *p*-value < 0.1.

HL Class 1	LL Class 2 (Engaged)		
	one-step	two-step	three-step
matern. educ.	−0.1588***	−0.1536***	−0.1610***
	(0.0417)	(0.0415)	(0.0432)
	LL Class 3 (Duty)		
	one-step	two-step	three-step
matern. educ.	0.1196***	0.1049**	0.0026
	(0.0415)	(0.0438)	(0.0478)
	LL Class 4 (Subject)		
	one-step	two-step	three-step
matern. educ.	−0.5551***	−0.5542***	−0.6117***
	(0.0582)	(0.0592)	(0.0714)
HL Class 2	LL Class 2 (Engaged)		
	one-step	two-step	three-step
matern. educ.	−0.0175	−0.0158	−0.0029
	(0.0434)	(0.0431)	(0.0482)
	LL Class 3 (Duty)		
	one-step	two-step	three-step
matern. educ.	0.1983***	0.1922***	0.1924***
	(0.0463)	(0.0494)	(0.0520)
	LL Class 4 (Subject)		
	one-step	two-step	three-step
matern. educ.	−0.2471***	−0.2517***	−0.3088***
	(0.0732)	(0.0745)	(0.0893)
HL Class 3	LL Class 2 (Engaged)		
	one-step	two-step	three-step
matern. educ.	−0.4458***	−0.4301***	−0.2059***
	(0.0741)	(0.0743)	(0.0782)
	LL Class 3 (Duty)		
	one-step	two-step	three-step
matern. educ.	−0.1262	−0.1316	−0.1444
	(0.1047)	(0.1080)	(0.1269)
	LL Class 4 (Subject)		
	one-step	two-step	three-step
matern. educ.	−1.1527***	−1.1487***	−1.2835***
	(0.1691)	(0.1911)	(0.2537)

In light of the similarity in the substantive results for the methods, we can consider the performance of the three-step methods acceptable even under these suboptimal class separation conditions.

## 7. Discussion

We have proposed a bias-adjusted three-step ML estimator for multilevel latent class analysis with covariates. In the first step, the single-level LC model without covariates is fitted to the data, ignoring the multilevel structure. In the second step, (lower-level) units are assigned to the classes on the basis of some classification rule, e.g., modal assignment. In the third step, the multilevel LC model with the covariates is estimated while correcting for classification error to obtain unbiased logistic parameter estimates for the association between class membership and covariates.

The performance of the three-step estimator was compared to the currently available modeling options for multilevel LCA with covariates, namely the one-step and two-step estimators, by means of a simulation study. Considering bias, the results showed that the three-step method is a viable alternative in most of the simulation conditions that were considered. Its performance is problematic when the higher-level separation is large and lower-level separation is small, but this bias is reduced substantially when the lower-level separation is at least moderate, and eliminated when the lower-level separation is high. When the higher-level separation is also moderate the three-step method performs as good as identical to the alternative estimators. Considering standard errors, the simulation study showed that the three-step method under-estimates them, which is a known feature of the method in single-level LCA (Bolck et al., 2004; Vermunt, 2010). The bias is less severe when the separation is larger on both the lower level and also larger on the higher level.

The findings for the performance of the three-step bias-correction method in multilevel LCA inform the ongoing debate about the choice between random-effect specifications and fixed-effect specifications. This debate has a long history in latent variable modeling (Aitkin, 1999; Aitkin & Alfó, 1998; Kankaraš et al., 2018) and beyond (for example in econometrics; see e.g., Peracchi, 2001). The reported performance of the random-effect three-step method under different combinations of lower-level separation and higher-level separation can serve as a practical indication of best-practice modeling approaches to applied multilevel LCA users. If the interest is in the lower-level structure, fixed-effect modeling may be a more appealing option when the higher-level separation is stronger relative to the lower-level separation.

In a real data example with moderate class separation at the lower level and between moderate and large class separation at the higher level, we compared the estimated covariate effects and their SEs for the three-step estimator and the alternative one-step and two-step estimators. Specifically, we identified citizenship norms among adolescents and analyzed its association with socioeconomic status. While the estimates were not identical, we observed that the three methods produced well-aligned substantial results, such that the three-step could be considered a legitimate modeling option even under these suboptimal conditions.

The current study is limited in the sense that we analyzed the performance of the proposed three-step method only for situations when the estimated model was correctly specified. One interesting avenue for future research is therefore to investigate the performance of the proposed method when modeling assumptions do not hold. In multilevel contexts, measurement non-invariance on the higher level, in which the item-response probabilities for the lower-level classes differ between higher-level classes, is likely to occur. This may lead to differences in the estimated response probabilities for the multilevel measurement model of interest and the step 1 single-level measurement model, thus introducing additional error in the step 2 class assignment. As such, it is recommended that future research look into controlling for measurement non-invariance on the higher level. For example, measurement non-invariance could be corrected for by means of fitting the multilevel measurement model and deriving the single-level model by marginalizing the conditional lower-level class proportions over the unconditional higher-level class proportions. Alternatively, measurement non-invariance could be corrected for by means of group (i.e., *j*-identifier) fixed effects (similar to Vermunt & Magidson, 2021).

Another topic for future research is the correction of the SEs of the covariate effects. In the single-level context, correction methods for the bias-adjusted three-step ML method have been presented by Bakk et al. (2014). The need for correction can be expected to depend on sample size and class separation at the lower level and the higher level. It would be worthwhile investigating how to extend these correction methods to the multilevel case and when the corrections are likely to be necessary in practice.

Finally, we have focused on the fully general parametrization of the multilevel latent class model. In applied research it is sometimes more appealing to adopt more constrained versions, such as random intercepts only and fixed slope parameters. While such models were outside the scope of the current study, it is nevertheless an interesting avenue for future research to examine the performance of the three-step under more constrained parametrizations.

## Footnotes

1 Replication files are available from the corresponding author upon request.

2 Replication files are available from the corresponding author upon request.
